# Intraoperative Near-Infrared Imaging Can Distinguish Cancer from Normal Tissue but Not Inflammation

**DOI:** 10.1371/journal.pone.0103342

**Published:** 2014-07-29

**Authors:** David Holt, Olugbenga Okusanya, Ryan Judy, Ollin Venegas, Jack Jiang, Elizabeth DeJesus, Evgeniy Eruslanov, Jon Quatromoni, Pratik Bhojnagarwala, Charuhas Deshpande, Steven Albelda, Shuming Nie, Sunil Singhal

**Affiliations:** 1 Department of Clinical Studies, University of Pennsylvania School of Veterinary Medicine, Philadelphia, Pennsylvania, United States of America; 2 Department of Surgery, University of Pennsylvania Perelman School of Medicine, Philadelphia, Pennsylvania, United States of America; 3 Department of Pathology, University of Pennsylvania Perelman School of Medicine, Philadelphia, Pennsylvania, United States of America; 4 Department of Medicine, University of Pennsylvania Perelman School of Medicine, Philadelphia, Pennsylvania, United States of America; 5 Departments of Biomedical Engineering and Chemistry, Emory University, Atlanta, Georgia, United States of America; Technische Universitaet Muenchen, Germany

## Abstract

**Introduction:**

Defining tumor from non-tumor tissue is one of the major challenges of cancer surgery. Surgeons depend on visual and tactile clues to select which tissues should be removed from a patient. Recently, we and others have hypothesized near-infrared (NIR) imaging can be used during surgery to differentiate tumors from normal tissue.

**Methods:**

We enrolled 8 canines and 5 humans undergoing cancer surgery for NIR imaging. The patients were injected with indocyanine green (ICG), an FDA approved non-receptor specific NIR dye that accumulates in hyperpermeable tissues, 16–24 hours prior to surgery. During surgery, NIR imaging was used to discriminate the tumor from non-tumor tissue.

**Results:**

NIR imaging identified all tumors with a mean signal-to-background ratio of 6.7. Optical images were useful during surgery in discriminating normal tissue from cancer. In 3 canine cases and 1 human case, the tissue surrounding the tumor was inflamed due to obstruction of the vascular supply due to mass effect. In these instances, NIR imaging could not distinguish tumor tissue from tissue that was congested, edematous and did not contain cancer.

**Conclusions:**

This study shows that NIR imaging can identify tumors from normal tissues, provides excellent tissue contrast, and it facilitates the resection of tumors. However, in situations where there is significant peritumoral inflammation, NIR imaging with ICG is not helpful. This suggests that non-targeted NIR dyes that accumulate in hyperpermeable tissues will have significant limitations in the future, and receptor-specific NIR dyes may be necessary to overcome this problem.

## Introduction

Surgery is the most effective therapy for solid tumors in the United States, and half of all cancer patients undergo surgery with curative intent.[1] However, despite a “curative” surgical resection, 20–50% of patients who undergo surgery develop local recurrences.[1] Patients who develop a local recurrence have a markedly reduced 5-yr survival.[1], [2]

Local recurrences are due to tumor cells that are left behind at the time of surgery. Small discrete tumors in solid organs can typically be removed with good results. On the other hand, defining the edges of the tumor (tumor margins) is particularly challenging in cancers that have invaded adjacent structures or have developed peritumoral changes due to vascular obstruction. These resections are more likely to be unsuccessful and to develop local recurrences. Surgeons typically use gross (macroscopic) examination of the tumor using visual inspection and finger palpation to define the tumor margins. However in many cases, this approach achieves tumor-negative surgical margins only 50% of the time.[3], [4] Surgeons can also utilize intraoperative pathology consultation. However, intraoperative frozen section presents its own set of difficulties including technical challenges of freezing tissues, tissue artifacts of freezing, cost, loss of tissue in smaller specimens for permanent section diagnosis, and lack of availability in “real-time”.

Many groups have begun investigating intraoperative near-infrared (NIR) imaging in order to identify tumors.[5], [6], [7], [8], [9], [10] NIR imaging is a low-energy approach, making it safe for the surgeon, patient, and surgical team. There are several NIR contrast agents, however, the only currently FDA approved dye is indocyanine green (ICG). ICG is well-tolerated and can be injected into patients for NIR cancer imaging.[11], [12], [13] It is not receptor-specific, but instead diffuses into tumors due to differences in vascular and lymphatic pressures.[5] ICG imaging is not possible for most diagnostic applications due to the lack of tissue penetration of the emitted light through the skin. However, when the body cavity is open, NIR imaging devices can detect ICG at depths of 10–15 mm in tissue.[14]

We hypothesized that NIR imaging using ICG may be able to identify tumors during cancer surgery. To test our hypothesis, we conducted a pilot study on several cancer models and human cases of solid tumors. We found that NIR imaging is a reasonable approach to identify tumors in solid organs. It allows for excellent contrast between normal tissue and cancerous tissue and is well-visualized intra-operatively. However, in situations where tumors develop surrounding inflammatory changes, NIR imaging is unable to discriminate non-cancerous from cancerous tissue.

## Materials and Methods

### Cell lines

The murine esophageal carcinoma cell line, AKR, was derived from mouse esophageal squamous epithelia with cyclin D1 over expression via Epstein-Barr virus ED-L2 promoter in p53 deficient genetic backgrounds and was a generous gift from Dr. Anil Rustgi (University of Pennsylvania).[15] The murine lung cancer cell line, TC1, was derived from mouse lung epithelial cells immortalized with HPV-16 E6 and E7 and transformed with the c-Ha-ras oncogene and was a generous gift from Dr. Steve Albelda (University of Pennsylvania).[16] The metastatic NSCLC cell line, murine Lewis lung carcinoma (LLC), was obtained from American Type Culture Collection (ATCC) (Manassas, VA). AE17 is an asbestos-derived murine mesothelioma cell line and was a generous gift from Dr. Steve Albelda (University of Pennsylvania [17]. EL4 was obtained from ATCC and is derived from a mouse lymphoma induced by 9,10-dimethyl-1,2-benzanthracene exposure. 4T1 also obtained from ATCC, is a metastatic murine mammary tumor line that is 6-thioguanine resistant.

Except for TC1 and AE17, cell lines were cultured and maintained in high-glucose DMEM (Dulbecco's Modified Eagle's Medium, Mediatech, Washington DC) supplemented with 10% fetal bovine serum (FBS; Georgia Biotechnology, Atlanta, GA), 1% penicillin/streptomycin, and 1% glutamine. TC1 and AE17 cell lines were cultured in RPMI (RPMI 1640 Medium, Mediatech, Washington DC) 10% FBS, 1% penicillin/streptomycin, and 1% glutamine. Cell lines were regularly tested and maintained negative for *Mycoplasma spp*.

### Reagents

Pharmaceutical grade indocyanine green (ICG) was purchased from Akorn Inc. (IC-GREEN, NDC 17478-701-02, Lake Forest, IL). C57bl/6 mice received 7.5 mg/kg ICG vial tail vein 16–24 hours prior to surgery.[6] Dogs and humans received 5 mg/kg ICG intravenous 24 hours before surgery.

### Near infrared fluorescent imaging systems

The hand-held near infrared imaging system has been previously described in detail.[18] In brief, a Raman Probe detector was incorporated into a cylindrical stainless steel sampling head integrated with a 5 m, two-fiber cable; one for laser excitation and the other for light collection. The sampling head and fiber cable were coupled via an FC connector to a spectrometer. The combined sampling head and spectrometer system has a wavelength range of 800–930 nm with 0.6 nm spectral resolution for near-infrared (NIR) fluorescence measurement. The excitation light was provided by a 785 nm, 100 mW continuous-wave diode laser. All readings were taken with a 0.1 second integration time and complied on a computer using proprietary software. This system was used for all canine studies, but only *ex vivo* in humans.

The optical NIR imaging device was developed in our laboratory.[19] Briefly, the device contains a 740 nm LED mounted on a heat sink. Emission spectra light from the tissue passes through a 780 nm bandpass filter into a CCD camera. A computer shows the images through PixeLINK Capture OEM software. The entire system is connected to a metal platform that can either be held in place via a ring stand or held by the surgeon using a handle adapter (developed by Mark Singer, BioMediCon©, Moorestown, NJ). Each imaged is captured twice, once in bright field and once in fluorescence. These images are processed and overlaid.

### Histochemistry

Tissues were harvested and bisected with one half either placed in Tissue-Tek OCT and stored at −80°C or in formalin for paraffin sectioning. To detect endothelial cells, monoclonal CD31 (mAB390) [20] was raised from hybridoma supernatant and purified. Frozen tumor sections were prepared as previously described.[21] CD31 expression was quantified by counting the number of positively staining cells in four high-powered (×400) fields.[22]

### Murine Studies

Female C57BL/6 (B6, Thy1.2) and BALB/c mice were purchased from Charles River Laboratories and Jackson Laboratories. All mice were maintained in pathogen-free conditions and used for experiments at ages 8 week or older. The Animal Care and Use Committees of the Children's Hospital of Philadelphia and the University of Pennsylvania approved all murine protocols in compliance with the Guide for the Care and Use of Laboratory Animals (Protocol 804894). Tumor cells for subcutaneous injections were suspended in 100 µL PBS. Tumor volume was calculated using the formula (π x long-axis x short-axis^2^)/6.

Surgery was performed on mice bearing flank tumors using an established partial resection model.[23] Surgery was performed when tumors reached ∼200 mm3. Mice were anesthetized with intramuscular ketamine (80 mg/kg) and xylazine (10 mg/kg), shaved, and the surgical field sterilized prior to surgery. Initially the mice were imaged to detect NIR signal and then subsequently a 1 to 2 cm incision was made adjacent to the tumor and the tumor was exposed using standard blunt dissection technique. After imaging, the incision was closed using sterile silk 4–0 sutures. Buprenorphine (0.2 mg/kg) was administered at the time of surgery and 6 hours postoperatively to provide analgesia. Preoperative treatment was unknown to the investigator performing surgery and making tumor measurements.

### Canine study design

Between June 2011 and April 2012, 8 consecutive dogs with primary lung tumors that were deemed surgical candidates were recruited from the University of Pennsylvania School of Veterinary Medicine. The canine study was approved by the University's Institutional Animal Care and Use Committee. The consent document was approved by the Veterinary School's Privately Owned Animal Protocol Committee and written informed consent was obtained from all owners (Protocol 802853). At the time of surgery, a standard-of-care thoracotomy and pulmonary resection performed. The affected lung lobe was removed using a surgical stapler (V3, Covidien, Mansfield MA).

During the operation, the intraoperative imaging system was used to inspect the chest before the pulmonary resection and after the pulmonary resection. The devices were draped with sterile plastic and were used to image the primary tumor *in situ*. Fluorescence readings were taken from the center of the tumor, grossly normal lung in the affected lobe or, in the case of large tumors, grossly normal lung in ipsilateral lobes. The tumor periphery was imaged in 4 radial directions, designated the 12, 3, 6, and 9 o'clock positions. Grossly normal lung 5 mm and 10 mm extending away from the tumor periphery was then imaged. The fluorescent margins were marked with suture.

Following removal of all tissues, the specimens were re-imaged *ex vivo* prior to submitting them to pathology. All tissues were then fixed in 10% formalin, embedded in paraffin, sectioned, and evaluated by a Board certified veterinary pathologist. The tumor margins, determined by fluorescence and marked with suture, were compared with histopathological margins.

### Human Studies

The human studies were approved by the Institutional Review Board (University of Pennsylvania School of Medicine, protocol 811870) and complied with the Board's required documentation and consent procedures. All patients underwent informed written consent before participating in this study. During surgery, the entire chest was palpated and visually inspected for disease. At that point, the camera system was sterilely draped and brought onto the surgical field for NIR imaging. Once the cancer resection was complete, the specimens were re-imaged on the back table of the operating room using the hand-held spectrometer. All specimens were sent for histopathology.

## Results

### NIR imaging can identify tumor deposits in normal tissue

Initially, in order to determine if a NIR contrast agent could identify solid tumors *in situ*, we conducted a proof-of-concept study in murine models of malignant disease. Fifty female C57bl/6 or BALB/c mice had one of five different syngeneic cancer cell lines (4T1 breast cancer, TC1 lung cancer, EL4 thymoma, AE17 mesothelioma, AKR esophageal cancer) injected into their flanks. After the tumors were well established (200 mm^3^), ICG was administered via the tail vein. The next day, a tissue spectrometer was used to semi-quantitate fluorescence from the tumor and surrounding organ.[18]

**Figure 1:**
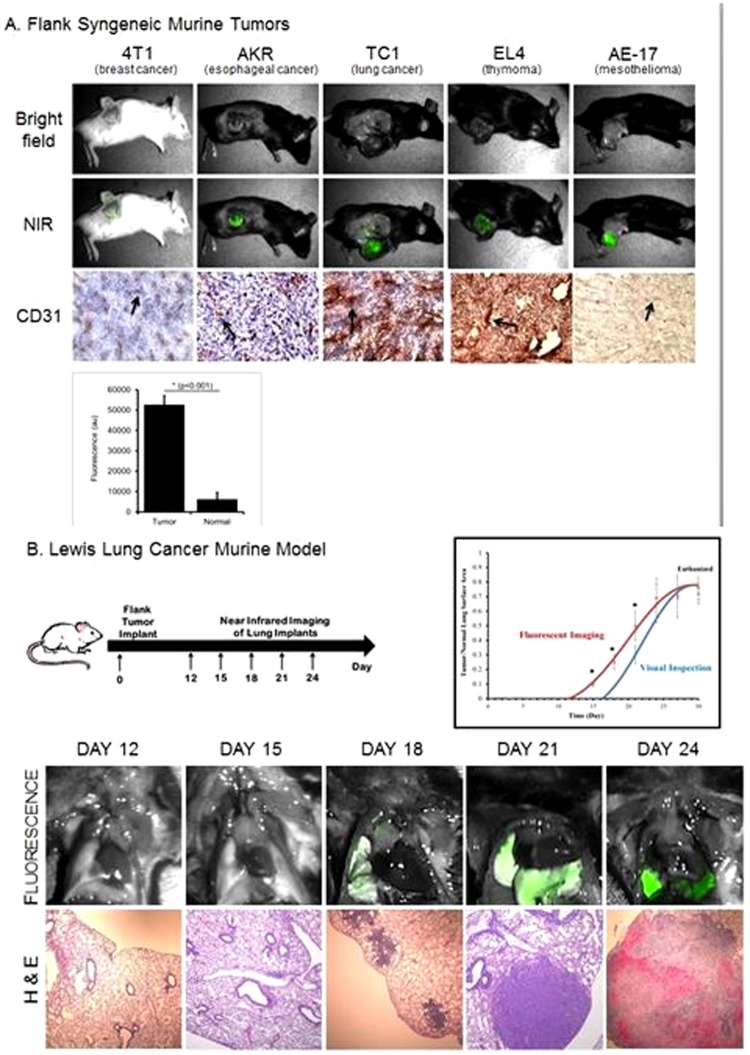
Preclinical evidence for NIR tumor labeling to detect primary and metastatic tumor deposits. (A) Five cancer cell types were injected into the flank of syngeneic mice. Once established (200 mm3), animals were dosed with 7.5 mg/kg of ICG and imaged. Tumors were harvested, imaged and stained for CD31 (Marked with black arrows). Histology images taken at 200x magnification. (B) C57bl/6 mice (n = 21) were injected with LLC cells in their flanks on Day 0. Starting on Day 12, the animals were euthanized, dosed with 7.5 mg/kg ICG 24 hours earlier and their thoracic cavities opened. Observers determined if the metastatic tumor nodules were visible in the lung. NIR imaging was then used to detect disease that was not visible to the un-assisted human eye. Histology images taken at 100x.

The mean fluorescence from the flank tumors was 52,710 arbitrary units (au) (range 46,283-60,000), and the mean fluorescence from surrounding normal tissues and organs averaged 6173 ± 3300 au. Thus, the mean signal-to-background ratio (SBR) was 8.5 (**Figure 1a**). We noticed that the fluorescence from different histological tumor subtypes was not markedly different. In the past, we and others have hypothesized that uptake of NIR dyes is variable and dependent on tumor vascularity.[5], [6] Thus, tumors of different histology should have different fluorescence. Tumors were harvested, sectioned, and assayed for microvessel density by CD31 staining. Although there was as wide range of microvessel density, we did not find a significant difference in the SBR of the tumor in different histological tumor types (p>0.1). In addition, we did not find that tumor fluorescence correlated with tumor vascularity (**Figure 1a**).

We then postulated that NIR labeling of tumors could detect cancer deposits of various sizes in the lung. C57bl/6 mice (n = 65) were injected into the flank (Day 0) with a murine cancer cell line, Lewis Lung Cancer (LLC), which spontaneously metastasizes to the lung. Every 3 days, mice (n = 3) were injected with 7.5 mg/kg of ICG via tail vein. The mice were euthanized, and the chests were opened and inspected for tumor burden. Flank tumors were imaged as before, and they were found to have a mean fluorescence of 53,290 ± 2668 au with a mean SBR of 10.8. Smaller versus larger flank tumors had little variability in fluorescence (p>0.1). We found that NIR imaging of the murine lungs could detect fluorescence from pulmonary metastases as early as Day 15. These deposits were not visible to the un-assisted eye and were as small as 0.2 cm by histology (**Figure 1b**). The mean tumor fluorescence in early small deposits under 2 mm was 39,923 ± 4577 au, well above the background fluorescence (mean 4290 au). These subcentimeter nodules had a mean SBR of 9.3. Metastatic pulmonary nodules became visible to the un-assisted eye by Day 24. The SBR of the tumors greater than 3 mm was 9.8, and not significantly different from the smaller tumors. However, the SBR of the tumors in the lung was lower than the SBR of the tumors in the flank. This was likely due to the technical challenges of imaging in the small cavity of the mouse chest and unrelated to actual tumor fluorescence.

In summary these data confirmed our hypothesis that NIR fluorescence from solid tumors can distinguish cancers from surrounding normal tissues. In addition, NIR imaging can identify small nodules with reasonable SBR and that this does not seem to depend on tumor vascularity.

### NIR imaging can identify tumors in solid organs in a naturally occurring canine lung cancer model

**Figure 2:**
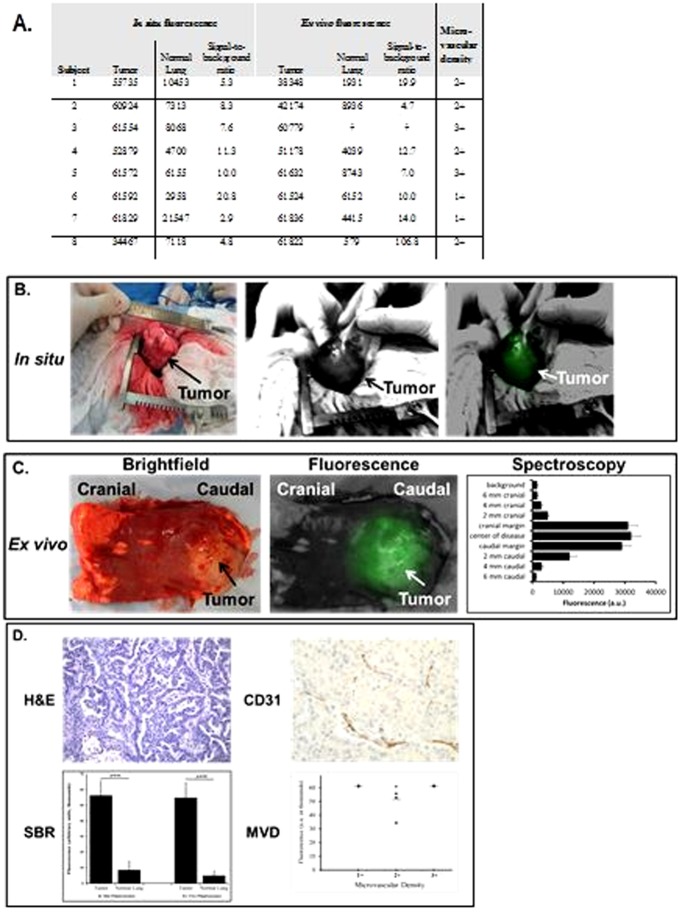
Representative intraoperative image of a canine lung cancer. (A) Signal-to-background ratio of tumor to surrounding normal lung tissue *in situ* and *ex vivo* in 8 canines. All values are reported in arbitrary units (a.u.). ^†^Due to the large size of this tumor, no measurements of normal lung fluorescence could be obtained ex vivo. (B) After opening the chest, the tumor was visualized in the chest. The tumor was well-circumscribed and was highly fluorescent (signal-to-background ratio 11.3). The tumor lies in the caudad position and the hilum of the lung is cranial. (C) *Ex vivo*, the tumor was fluorescent (SBR 12.7) and the margins of the tumor were well-defined. (D) H&E confirmed a lung adenocarcinoma with 2+ CD31 staining. The signal-to-background ratio (SBR) was higher *ex vivo* than *in situ* because of superior control of the surrounding environment such as lighting conditions, exposure and lack of motion. Although the fluorescence from the tumors did not significantly change, the background fluorescence from the normal lung was reduced when the environment could be better controlled. Microscopically, the tumor microvascular density (MVD) did not seem to impact the degree of fluorescence.

Small animal imaging is conducted in a controlled, artificial environment, so we evaluated NIR imaging for identification of solid tumors in a rigorous large animal model in the clinical setting. Eight outbred canines with naturally occurring tumors who presented to the University of Pennsylvania School of Veterinary Medicine surgery clinic with a primary lung tumor were enrolled in the study with informed consent from their owners and institutional approval (**Figure 2a**). Ages ranged from 4 to 14 years (median 10 years) and weights ranged from 6 to 60 kg (median 24 kg) (**Table 1**). Three dogs were spayed females and 5 dogs were castrated males. All dogs received ICG via the cephalic vein without any adverse reactions 24 hours before surgery.

**Table 1:** Canine characteristics.

Subject	Breed	Age (years)	Weight (kg)	Pathology	Tumor size (cm)	Operation performed
1	Labrador retriever	11	60	adenocarcinoma	6	Left caudal lung lobectomy
2	Australian shepherd	10	15	adenocarcinoma	4	Right caudal lung lobectomy
3	Giant schnauzer	10	44	neuroendocrine	15	Left caudal lung lobectomy
4	Cocker spaniel	11	13	squamous cell carcinoma	6	Right caudal lung lobectomy
5	Bulldog	8	32	adenocarcinoma	5	Left caudal lung lobectomy
6	Miniature pinscher	14	6	adenocarcinoma	6	Left caudal lung lobectomy
7	Miniature schnauzer	9	7	adenocarcinoma	2	Thoracoscopic assisted partial lobectomy
8	Labrador retriever	4	35	adenocarcinoma	8	Right cranial lung lobectomy

At surgery, in order to determine if NIR imaging could identify lung cancers from the surrounding tissues, all canines underwent imaging of the tumor and normal lung. The average tumor size was 6.5 cm (range 2–15 cm). *In situ*, the primary tumor could readily be pinpointed in the lung by NIR imaging (**Figure 2b**). Three spectral readings were taken at the center of the tumor and in 6 regions around the rim of the tumor. The fluorescent intensity of the majority of tumors ranged from 30,000 to 60,000 au. The lung parenchyma was imaged at multiple locations to establish a background spectral emission value. Most of the variability occurred in the normal background lung parenchyma because the spectral emission was low (less than 5000 au) and difficult to quantitate. The mean tumor SBR was 8.8, and the tumor SBR ranged from 2.9 to 20.8, thus, it was easy to distinguish tumor from normal tissue by NIR imaging. When we examined intra-tumoral variability from the center of the tumor to the rim of the tumor, there was minimal difference (p>0.4). The surgeon was able to look at the NIR images real-time during the case and rapidly identify the tumor from normal tissue. All 8 tumors appeared equally fluorescent to the surgeon, and he could not identify which tumors had a low fluorescence (ie. SBR 2.9) versus a high fluorescence (ie. SBR 20.8).

We found that there was substantial variability in tumor fluorescence depending on the ambient light conditions, ability to position the spectrometer and access to the entire tumor in the animal. In order to obtain more standardized readings, the tumors were resected from the dog and re-imaged on the back table of the operating room. *Ex vivo*, the mean tumor SBR was 11 fold higher than the surrounding lung parenchyma (p = 0.016) (**Figure 2c**). There was significantly less intra-variability in fluorescence in the tumor once the lighting conditions and positioning of the spectrometer could be better controlled on the back table. We removed an outlier (Subject #8) from this calculation due to the strong signal from the tumor (100+ fold difference signal-to-background ratio between tumor and normal lung). On average, we found the *ex vivo* image had a higher SBR compared to the *in situ* image (*in situ* SBR 8.8 versus *ex vivo* SBR 11.4) because the background noise was reduced, not because the tumor was more fluorescent (**Figure 2d**).

In order to determine if the fluorescence of the tumor correlated with tumor vascularity, we compared the SBR of the tumor to the microvascular density (MVD). All primary tumors were stained for CD31. Two independent investigators categorized the tumors as 0, 1+, 2+ and 3+ MVD. There was a wide range of fluorescence based on the hand held spectrometer both *in situ* and *ex vivo*, and given the small sample size, we did not see any correlation between vascular density and degree of fluorescence (**Figure 2d**). Also, we could not evaluate the impact of tumor size on fluorescence due to the lack of sufficient sample size.

### NIR imaging cannot distinguish cancer from atelectasis at the tumor margins

As tumors grow and develop into a major burden in the host organ, peritumoral inflammation can occur due to the mass effect on the organ's blood supply and venous drainage. For example, pulmonary lobes will frequently develop obstructive pneumonitis and congestion from a large tumor blocking the bronchus and venous drainage to a lung segment.[24] This has important implications. In humans, if a tumor causes distal obstruction, the prognosis is worse and the patient is designated to have T2 lung cancer.[25] During an operation, surgeons often cannot distinguish tumor tissue from surrounding inflammatory changes by palpation and visualization. Thus, we wanted to determine if NIR imaging could discriminate abnormal peritumoral congestion from normal tissue and cancer, and whether NIR imaging was superior to the surgeons' ability to identify cancer from inflammation at the margins.

First, we compared the sensitivity of locating the edge of the tumor by the surgeon versus the NIR device. The lung carcinoma was first palpated by the surgeon and blue stitches were placed circumferentially to mark the surgeons' assessment of the tumor margins. Then, the NIR camera was used to identify the tumor margins. If there was a discrepancy between the surgeons' assessment of the tumor edge and the fluorescence, another marking stitch was placed in the new margin. Spectral emissions were recorded at each location.

**Figure 3:**
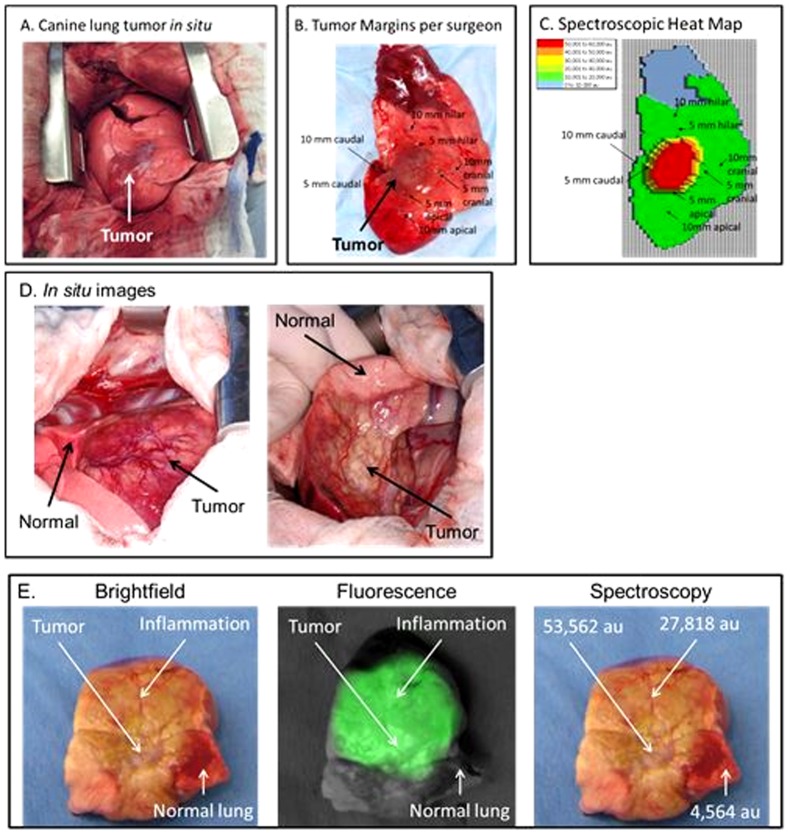
Intraoperative imaging of surgical margins during pulmonary resection. (A) *In situ*, the tumor can be visualized and palpated. (B) Stitches mark the tumor margin at 5 mm intervals from the palpable tumor edge. (C) The spectrometer was used to measure NIR fluorescence (805 nm) at each location on the specimen and develop a heat map. The heat map predicted the tumor margins assessed by the surgeon and the pathologist. In a second case with significant peritumoral inflammation, (D) intraoperative images demonstrated a tumor in a pulmonary lobe as it was retracted from the canine chest. (E) *Ex vivo*, the pulmonary lobe could be seen fluorescing, however, it was difficult by brightfield or fluorescence to discriminate the margins between tumor tissue and inflammatory lung tissue. Spectroscopy demonstrated some difference in the fluorescence from the tumor versus congested tissue, but clinically this was tedious and not practical. The surgeon also had difficulty in identifying tumor from non-tumor tissue by manual palpation because the lung was congested and edematous.

In 5 canines, the tumors did not have any post-obstructive pneumonitis, thus there was no significant fluorescence beyond the rim of the tumor. In these cases, the tumor margins were palpable by the surgeon and similar to the margins identified by NIR imaging (**Figure 3a**). The edge of the tumor was fluorescent (mean 51,324 au), and the tissues within 5 mm of the edge of the tumor was markedly different. Within 5 mm away from the tumor, the lung parenchyma was no longer fluorescent (mean 12,642 au) and the SBR decreased from greater than 8 to less than 1.5. Serial sectioning by the pathologist confirmed that both the surgeon and NIR imaging could identify the rim of the tumor within 2 mm.

**Figure 4:**
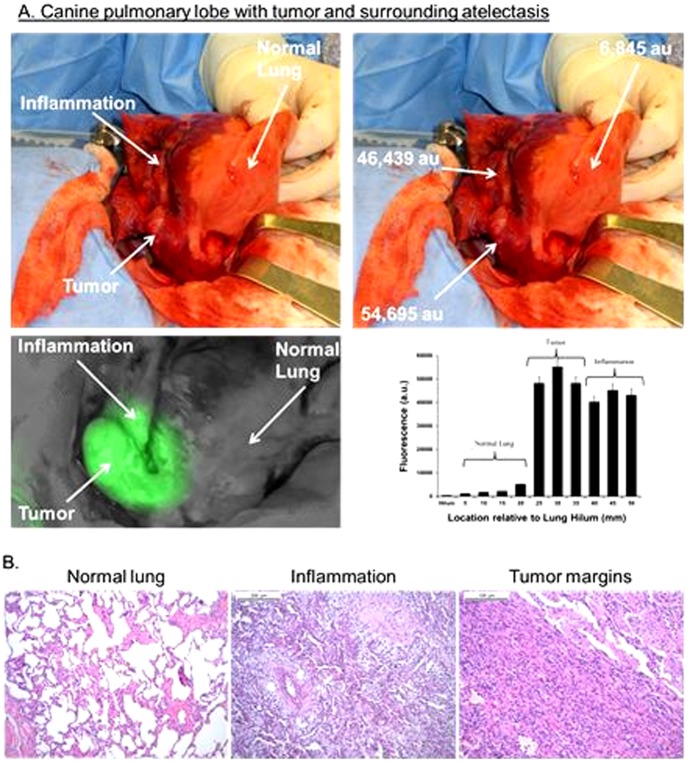
Intraoperative imaging of surgical margins during pulmonary resection. (A) Intraoperatively, the animal underwent right thoracotomy and palpation of the primary tumor. Intraoperative image of the pulmonary lobe as it is retracted from the canine chest revealed the dorsal portion of the lobe has significant compression (ie. atelectasis) from tumor obstruction. The ventral portion of the lung retained its normal appearance. Spectroscopic analysis of the tumor was performed *in situ*. All sites were recorded in triplicate and averaged. The graph shows the inflammatory tissue was highly fluorescent and could not be distinguished from tumor. (B) H&E demonstrated normal alveolar parenchyma (left panel), congestion lung with neutrophils and fibrotic plugs (middle panel) and tumor (right panel) from representative biopsies of the pulmonary lobe.

In 3 canines, the tumors were large (6, 8 and 15 cm) and they resulted in venous congestion, obstructive pneumonitis and collapse of the tissues surrounding the cancer. In these cases, we found significant variability in the fluorescence in the abnormal tissue surrounding the tumor. These abnormal tissues emitted high fluorescence (**Figure 3b**). The difference in fluorescence between the tumor edge (mean SBR 8.4) and 5 mm from the tumor edge (mean SBR 7.9) was not markedly different. Even at 10 mm away from the tumor edge, the mean SBR was as high as 5.7. We used the spectrometer to try to improve discrimination of the SBR, however, this did not improve our ability to tell apart tumor from inflammation. Histologically, the areas of inflammation did not have tumor cells. They had distorted tissue architecture, macrophages, edema, and in some cases massive neutrophil infiltration and necrosis. When the surgeon looked at the optical images, he could not distinguish tumor from atelectasis or inflammation based on fluorescence (**Figure 4**).

Together, these data demonstrated that NIR imaging can accurately distinguish margins in lung tumors compared to surrounding normal lung parenchyma but cannot identify margins accurately in lung tumors that have peritumoral inflammation. NIR imaging was not particularly superior to the surgeons' ability to tell apart normal versus cancerous tissue.

### NIR imaging can identify solid human tumors

To determine if human tumors can be distinguished from normal surrounding tissues by NIR imaging, we enrolled 5 patients undergoing surgery for removal of their cancer (3 lung nodules, 1 chest wall mass and 1 anterior mediastinal mass). Their ages ranged from 49 to 69 years (median 62 years). Two surgeons reached a consensus about the clinical stage and operative approach prior to surgery. All enrolled patients were thought to have limited disease, amenable to surgery, and no metastases (ie. potentially curable). The median tumor size was 2.3 cm (range 1.8–9.1 cm) on preoperative imaging. Patients were injected with ICG prior to surgery. At the time of surgery, the body cavity was opened and inspected. In all cases, the surgeon could immediately visualize or palpate the tumor. The patients underwent a standard-of-care surgical resection of the tumor.

Once removed from the patient, the specimen was examined, opened, biopsied and analyzed *ex vivo*. Qualitatively, NIR imaging revealed strong fluorescence in all masses. The hand held spectrometer was used to semi-quantitate tissue fluorescence. Each tumor had 4 measurements at four perpendicular locations and the center of the tumor (total of 20 measurements/tumor). Mean fluorescence in the human tumors was 51,756 ± 2266 au with an SBR of 8.1. The fluorescence from the tumors was remarkably homogeneous throughout the tumor.

**Figure 5:**
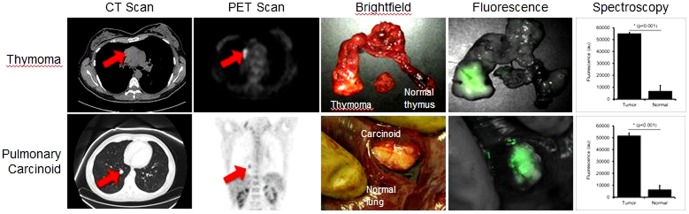
Two representative human tumors. Computed tomography (CT) scan and positron emission tomography (PET) scan demonstrated an anterior mediastinal mass and a lung nodule in two patients. Patients were injected with ICG, and then underwent resection of their tumors. *Ex* vivo, NIR imaging demonstrated the tumors were highly fluorescent and the surrounding organ had minimal background noise. The optical images were easy to interpret by the surgeon and facilitated the identification of the tumor. Spectroscopy demonstrated a SBR of 8.1 for the thymoma and 7.9 for the carcinoid. The tumors were discrete and well-circumscribed and had minimal peritumoral inflammation.

In 4 cases, the tumors were relatively small (range 1.8–3.1 cm), and there did not appear to be any peritumoral inflammation or collapse. The average signal diminished from over 50,000 au at the tumor margin to less than 20,000 au within 5 mm of the gross tumor margin. The tumor margins were easy to visualize and finger palpate by the surgeon. Using the NIR imaging device, the fluorescence from the tumor could be readily distinguished by the surgeon (**Figure 5**).

**Figure 6:**
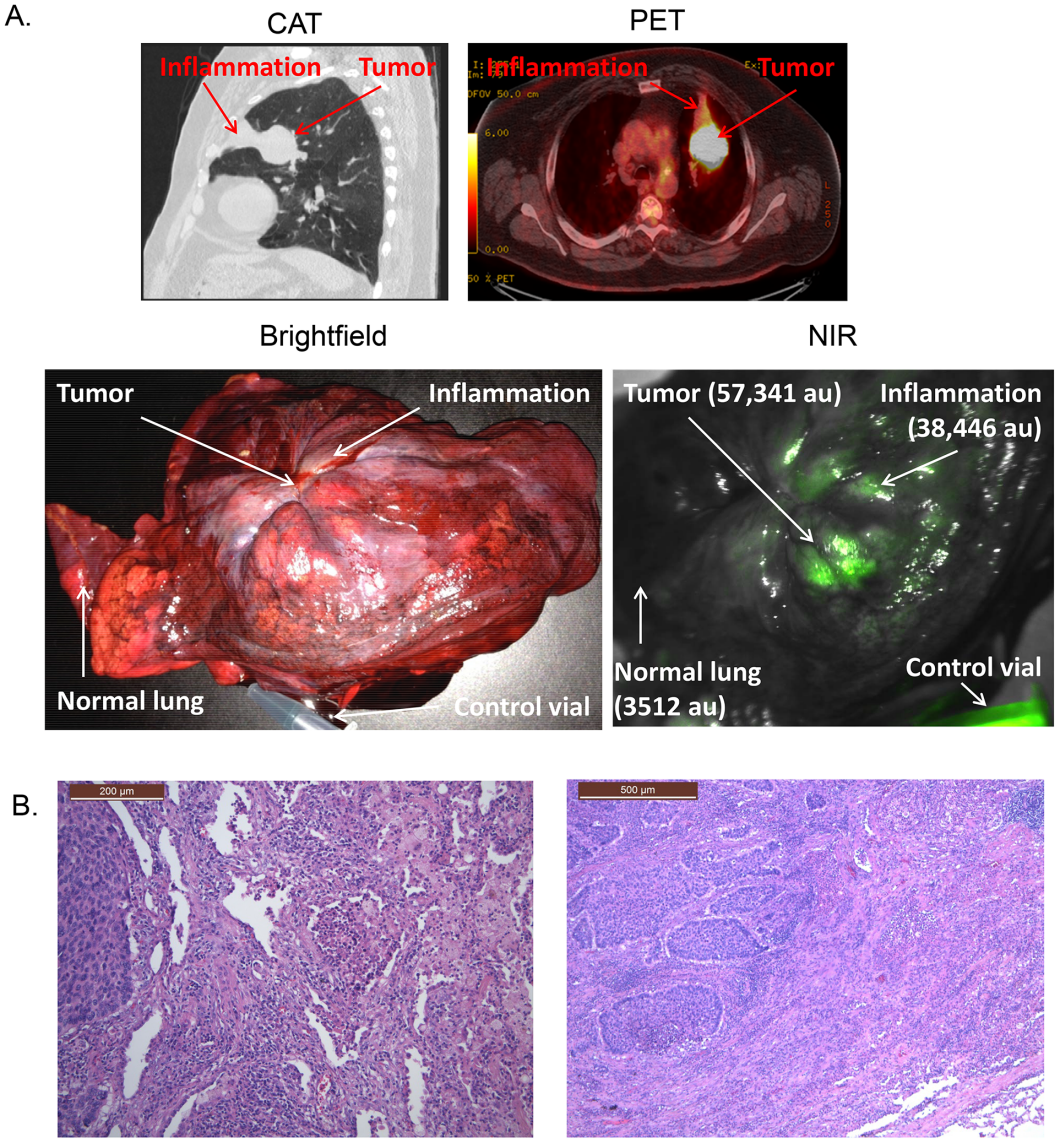
Representative photos of a patient with an 8 cm left upper lobe squamous cell carcinoma of the lung. (A) The CAT scan and PET scan demonstrate the tumor in the periphery of the lung with surrounding post-obstructive collapse of the lung. Once the tumor was removed, it is hard to distinguish tumor from inflammation in the left upper pulmonary lobe. NIR imaging shows similar fluorescence from both areas. (B) Histologically, the areas of inflammation that fluoresced did not contain tumor cells but had congestion, edema, fibrotic plugs and inflammatory cells.

In one case, the tumor was large (9.1 cm) and the lung surrounding the tumor was obstructed. The tumor SBR was 8.9. The surgeon marked the edge of the tumor by finger palpation. On NIR imaging, however, the fluorescence extended beyond the edge of the suspected margin into the atelectatic lung (SBR 5.6) (**Figure 6**). The tumor, surrounding lung and a section of unobstructed normal lung were then sectioned by the pathologist. The tumor margin identified by the surgeon was not accurate, and there were tumor cells beyond the marked margin. NIR imaging, however, was also not accurate. It identified a large section of atelectatic lung that was obstructed and congested, but did not contain tumor cells. When the tumor and region surrounding the atelectatic lung was cross-sectioned by the pathologist, extensive tissue collapse and venous congestion could be grossly seen at the rim of the lung cancer.

In summary, in this pilot study, no patients were lost to follow up, and there were no obvious toxicities, adverse events or deaths related to injection of ICG. Final pathology on the 5 cases demonstrated lung cancer (n = 2), carcinoid (n = 1), thymoma (n = 1), and chest wall sarcoma (n = 1). All patients were eligible for the analysis and were alive at the submission of this manuscript. ICG was able to distinguish abnormal tissues from normal tissues but could not discriminate inflammatory tissue from cancer.

## Discussion

In this report, we describe an approach to image solid tumors using a FDA approved near-infrared (NIR) fluorophore, indocyanine green (ICG). We found that this technology could be used during surgery to identify solid tumors as small as 1 mm in mice. The most important finding in this work was our ability to label a wide array of canine and human tumors *in situ* by systemic delivery of ICG. NIR imaging was exquisitely sensitive for tumors, and tumor fluorescence did not seem to relate to the degree of tumor vasculature. Others have had similar success imaging breast cancer and colorectal cancer metastatic to the liver.[26], [27]

One proposed application of intraoperative NIR imaging is to identify tumor from non-cancerous tissues. In small discrete tumors, the tissue surrounding the cancer is typically not congested and has well-defined borders. In these situations, NIR imaging was effective in discriminating the tumor from the surrounding tissue. The surgeon was able to finger palpate the tumor and locate these margins. For larger tumors, the surgeon had difficulty in distinguishing tumor from invasion and/or inflammation. Unfortunately, NIR imaging was not helpful for defining tumor margins in these situations. For these tumors, inflammation and venous congestion surrounding the tumor fluoresced and was a major limitation to NIR imaging.

This finding reflects the fundamental clinical limitations of ICG drug delivery.[28], [29] ICG is avidly taken up in tissues that have “leaky capillaries” due to the enhanced permeability and retention (EPR) effect.[5] Upon injection into the blood, ICG quickly binds to proteins (albumin, lipoproteins), and the resulting ICG-protein complex is 4–6 nm in size. This small size allows preferential targeting and prolongs the plasma half-life of the tracer. The ICG-protein complex then accumulates preferentially in tumor tissue due to the presence of defective endothelial cells, wide fenestrations (up to 4 µm), and paucity of lymphatic drainage.[30] However, ICG is not receptor-specific, therefore, it does not preferentially collect in only tumor tissues.

Both tumor and inflammatory tissues have similar vascular changes in their microenvironment.[31] Tumors require neoangiogenesis to maintain nutrient and oxygen supplies to rapidly dividing cells. Tumor vasculature is disordered with blood vessels that are abnormal both morphologically and functionally. The vessels are often distended, asymmetrical in distribution and contour, and have irregular pericyte and basement membrane coverage. Similarly, vascular hyperplasia is common in reactive and inflammatory tissues.[32] There is increased proliferation of endothelial cells, perivascular mast cells and microvascular density. Thus, ICG is equally likely to accumulate in tumors and inflammatory tissues.

One major difference we discovered between human and canine lung cancer is the difference in tumor size at presentation. In humans, lung cancers are often detected when they are small and asymptomatic. However, canine lung tumors do not become noticeable until the animal becomes ill or symptomatic (i.e. hemoptysis or cough). In this series, the canine tumors ranged in size from 2 to 15 (median 6) cm. Therefore, canine lung tumors tend to be larger at presentation with a significant area of surrounding atelectasis and obstructive pneumonitis.

Whether false positive fluorescence from inflammatory tissues is clinically relevant is debatable. In many situations, the inflammatory tissue is likely to require resection because it has lost its function and will remain a detriment to the patient. For lung cancer, surgeons typically perform a wide excision beyond the tumor, thus the abnormal tissue is expected to be removed. However, this inability to differentiate inflammatory tissues from cancer may be more relevant and have a major impact in clinical decision making in other tumor types.

It should be noted that we used the NIR spectrometer and the CCD imaging system interchangeably in this study.[17], [18] In general, the spectrometer was advantageous due to its ability to provide quantitative data. However, the device is time-consuming, and cannot be used in vivo in humans (not FDA approved). The CCD camera provides useful 2-dimensional images for real-time application during surgery, but it lacks quantitative information.

In the future, targeted NIR dyes are likely to emerge for clinical use. Several agents are currently being studied in preclinical models.[33], [34], [35] These contrast agents will overcome the challenge of non-selective uptake in inflammatory tissue. Receptor-targeted NIR contrast agents will likely accumulate several fold higher in tumor tissues. Although ICG may be too non-specific for some clinical applications, our data do show that intraoperative NIR is feasible from a logistical and technical point of view.
